# Probiotics in the prevention and treatment of calcium oxalate kidney stones: mechanisms and therapeutic potential

**DOI:** 10.3389/fmicb.2025.1663138

**Published:** 2025-10-02

**Authors:** Jinshan Yang, Dengbao Li, Tao Li, Benzhong Jia

**Affiliations:** ^1^College of Clinical Medicine, Guizhou Medical University, Guiyang, China; ^2^Department of Urology, Affiliated Hospital of Guizhou Medical University, Guiyang, China

**Keywords:** probiotics, kidney stones, calcium oxalate stones, gut microbiota, oxalate degradation, recombinant probiotics

## Abstract

**Background:**

Kidney stones, particularly calcium oxalate stones, remain a significant health issue despite advances in treatment techniques. Residual stones that persist after treatment can lead to recurrent stone formation and further complications. The role of gut microbiota, specifically probiotics, in modulating oxalate metabolism has gained increasing attention as a potential therapeutic strategy to reduce residual stones and prevent recurrence.

**Summary:**

This paper reviews the potential of probiotics, including recombinant strains, in the treatment of calcium oxalate kidney stones. Probiotics are thought to promote the degradation of oxalate in the gut, thereby reducing its absorption and preventing stone formation. Recent studies have highlighted the beneficial effects of probiotic interventions on gut microbiota composition, oxalate degradation pathways, and calcium oxalate stone formation. Moreover, recombinant probiotics, engineered to enhance oxalate-degrading capabilities, hold promise for improving treatment outcomes.

**Key messages:**

This review summarizes recent advancements in the use of probiotics for the prevention and treatment of calcium oxalate kidney stones, with a focus on their role in oxalate degradation and the potential of recombinant probiotics to improve treatment outcomes.

## 1 Introduction

Kidney stone disease is a prevalent condition affecting the urinary system. The global morbidity and prevalence of nephrolithiasis are steadily increasing, with recent data indicating a prevalence of 12% in men and 10% in women (Singh et al., 2022). The formation of kidney stones is believed to be closely associated with various factors, including diet, genetics, and metabolism (Khan et al., 2016). Although the mechanisms underlying kidney stone formation remain unclear, urine supersaturation and crystallization are considered the primary drivers of renal crystal deposition (Wang Z. et al., 2021). Kidney stones are classified based on their composition, which includes calcium oxalate, uric acid, phosphate, and cystine stones, with calcium oxalate accounting for approximately 80% of cases (Coello et al., 2023). Calcium oxalate stones are characterized by high incidence and recurrence rates, leading to complications such as ureteral obstruction, frequent urinary tract infections, painful urination, and, if left untreated, kidney damage (Ziemba and Matlaga, 2017).

Current treatment options for calcium oxalate kidney stones primarily include surgery, medication, and lifestyle modifications. However, these treatments have limitations, including the invasive nature and high recurrence rates of surgical interventions, as well as significant side effects associated with medications (Zhang et al., 2023). Therefore, it is critical to explore safer and more effective methods for preventing and treating calcium oxalate nephrolithiasis. Recent advancements in technologies such as high-throughput sequencing have suggested that probiotics may influence the occurrence and recurrence of calcium oxalate stones, although the specific mechanisms remain unclear (Kelsey, 2016). Understanding the potential effects of probiotics on calcium oxalate nephrolithiasis could provide valuable insights into the pathogenesis of nephrolithiasis and open new avenues for its prevention and treatment.

## 2 Probiotics and the “gut-kidney axis”

**Figure 1:**
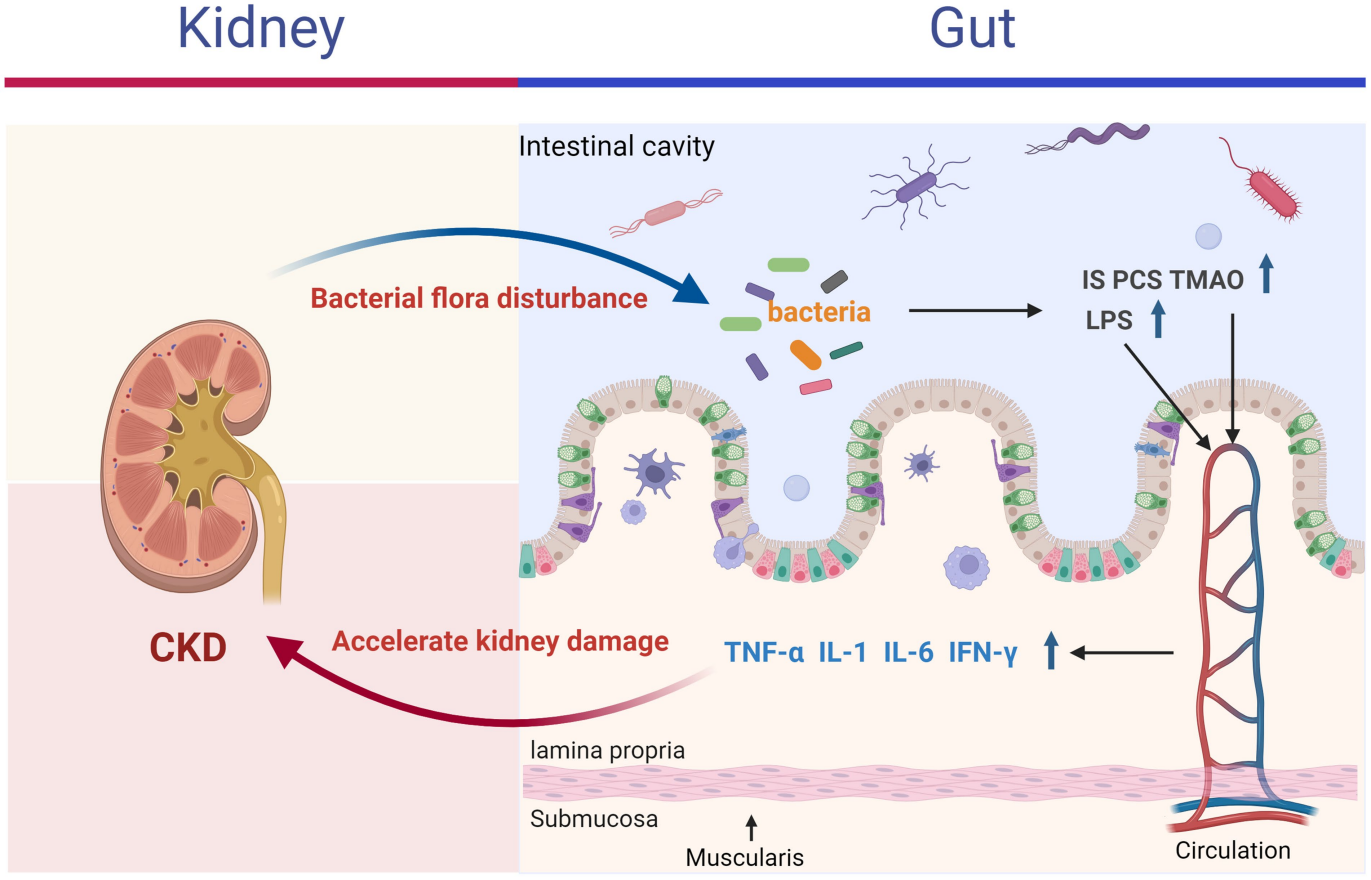
Two-way interaction between the kidneys and the intestines.

The “gut-kidney axis” theory posits that alterations in the gastrointestinal tract and kidneys can mutually influence each other, leading to adverse outcomes through mechanisms such as energy metabolism, immune inflammation, and interactions with gut microbiota (Ni et al., 2022). Increasing evidence indicates that conditions like inflammatory bowel disease, chronic kidney disease, kidney stones, and uremia are associated with the gut-kidney axis (Colella et al., 2023). According to this theory, kidney disease can disrupt the balance of intestinal microbiota, promoting the proliferation of enteric pathogens and the production of enterogenic urotoxins (Meijers and Evenepoel, 2011). These toxins may accumulate in the kidneys, exacerbating the decline in kidney function (Wing et al., 2016; Meijers et al., 2018). Conversely, disruptions in intestinal homeostasis can impair the gut barrier, allowing bacteria, bacterial components (e.g., endotoxins such as lipopolysaccharides), and urotoxins to enter the bloodstream, thereby triggering systemic inflammation through the release of inflammatory mediators, including IL-1, IL-6, TNF-α, and IFN-γ (shown in Figure 1) (Ghosh et al., 2020; Foresto-Neto et al., 2021).

Recent research on the gut-kidney axis has spurred the development of probiotic-based strategies aimed at preventing and treating calcium oxalate kidney stones. Probiotics, defined as live microorganisms that confer health benefits to the host, can colonize the gastrointestinal and reproductive systems, enhancing immune function by maintaining a balanced gut microbiota. The diversity and abundance of the gastrointestinal microbiome are closely linked to overall human health (Wang X. et al., 2021; de Vos et al., 2022). Kim et al. (2022) collected fecal samples from 915 adults, dividing them into three groups, and performed 16S rRNA gene sequencing to examine the diversity and taxonomic characteristics of gut microbiota associated with kidney stone status. Their findings indicated that kidney stones were linked to alterations in gut microbiota compared to the control group. Probiotics, a crucial component of gut microbiota, have been suggested to encompass various species, including *formic acid bacteria*, *Lactobacilli*, *Bifidobacteria*, and *Enterobacteria*, which are believed to play roles in the prevention and treatment of calcium oxalate kidney stones (Wigner et al., 2022). Yuan et al. (2023) further demonstrated that oral probiotic preparations, whether administered alone or in combination, including oxalate-degrading bacteria such as *Oxalobacter formigenes*, *Lactobacilli*, *Bifidobacteria*, and *Enterococci*, can reduce urinary oxalate excretion in both humans and animal models. Therefore, probiotics for the treatment and prevention of renal calcium oxalate stones may represent a promising new approach.

## 3 Effect of oxalic acid probiotics on renal calcium oxalate stones

### 3.1 Oxalobacter formigenes

**Figure 2:**
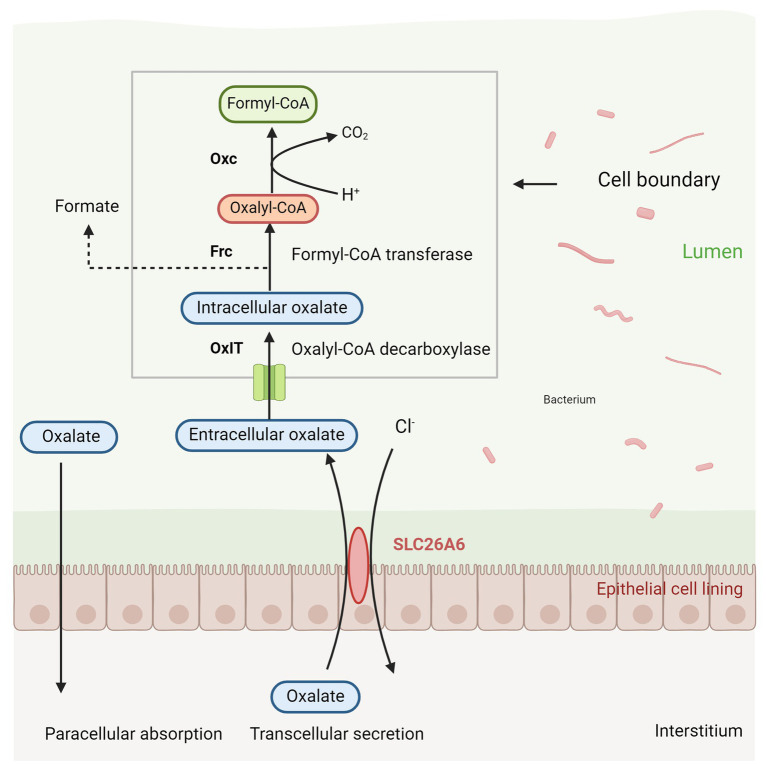
Oxalate metabolism by *Oxalobacter formigenes*.

*Oxalobacter formigenes* is a Gram-negative, anaerobic bacterium that primarily degrades oxalic acid, serving as a major carbon source within the intestinal microbiota (Ellis M. E. et al., 2016). This bacterium plays a critical role in regulating the availability of oxalates in the intestinal lumen, thereby protecting against hyperoxaluria and preventing the formation of calcium oxalate stones (Daniel et al., 2021). Hyperoxaluria is a significant risk factor for the development of calcium oxalate stones, with approximately half of urinary oxalate originating from the gut. Consequently, limiting intestinal oxalate absorption is crucial for reducing the risk of stone formation or recurrence (Chen et al., 2023). Siener et al. (2013b) demonstrated that colonization by *Oxalobacter formigenes* reduces intestinal oxalate absorption, leading to decreased urinary oxalate excretion, as evidenced by fecal samples from patients with calcium oxalate stones. The bacterium’s mechanism for oxalate degradation involves the use of an oxalate-formate antiporter (OxlT) to transport extracellular oxalate into the cell, where it is converted into oxalyl-CoA by formyl-CoA transferase (Frc). Oxalyl-CoA is subsequently decarboxylated into formate and CO2 by oxalyl-CoA decarboxylase (Oxc), with formate being exported out of the cell via OxlT, thereby completing the degradation process (shown in Figure 2) (Youssef, 2024). Additionally, *Oxalobacter formigenes* may enhance the exudation of plasma oxalate into the gut by modulating the expression of the intestinal anion exchanger solute carrier family 26 member 6 gene (SLC26A6) (Stepanova, 2023).

Jafari et al. (2021) analyzed urine and fecal samples from 73 patients with calcium oxalate urolithiasis and found a significant negative correlation between the presence of *Oxalobacter* species and the formation of calcium oxalate stones. Earlier studies using rat models of primary hyperoxaluria demonstrated that *Oxalobacter formigenes* intervention reduced crystal formation in the kidneys (Verhulst et al., 2022). In a case–control study, Kim et al. (2021) found that colonization by formic acid-producing *Oxalobacter* reduced the risk of recurrent calcium oxalate stones by nearly 70% in 47 recurrent stone formers compared to 259 controls. Similarly, Hiremath and Viswanathan (2022) reported that 70% of kidney stone patients who were not colonized by *Oxalobacter formigenes* showed a noticeable increase in plasma oxalate after following a standardized diet. In addition, a phase III clinical trial conducted by Milliner et al. (2018) demonstrated that, compared with placebo, treatment with *Oxalobacter formigenes* did not result in a significant reduction in urinary oxalate levels. Other studies have reported no significant difference in urinary oxalate excretion between patients who tested positive or negative for *Oxalobacter formigenes* (Siener et al., 2013b). Such findings may be attributed to insufficient colonization, population heterogeneity, or inadequate treatment duration in these studies. Collectively, these findings suggest the potential use of *Oxalobacter formigenes* in preventing renal calcium oxalate stones.

Despite its promise, *Oxalobacter formigenes* has limitations, including its low tolerance to aerobic conditions and inhibition by bile salts and acidic environments, which complicate its colonization in the gut (Ellis M. L. et al., 2016). Studies have also indicated that *Oxalobacter* colonization is transient and may depend on the luminal concentrations of calcium, oxalate, and pH (Hatch et al., 2011). Furthermore, Nazzal et al. (2021) demonstrated that antibiotics, used in *Helicobacter pylori* eradication trials, significantly suppressed *Oxalobacter* colonization. The bacterium’s dependence on oxalate may also limit its efficacy in preventing other types of kidney stones. These factors pose challenges to its use as a probiotic for the prevention of calcium oxalate stones.

### 3.2 *Lactobacillus* species

*Lactobacillus* is a genus of Gram-positive, non-spore-forming bacilli abundant in the human gut, typically considered facultatively anaerobic with fermentative metabolism. Over fifty species of *Lactobacillus* are known to persistently colonize the gastrointestinal tract of healthy individuals (Rossi et al., 2016). Although present in smaller quantities, these bacteria are closely related to human health (Ren et al., 2020). Due to their generally safe profile, *Lactobacilli* are commonly used as probiotics.

The potential role of *Lactobacilli* in oxalate degradation and the prevention of calcium oxalate kidney stones has gained attention in recent years (Martín and Langella, 2019). Soliman et al. (2021) isolated 88 strains of *Lactobacillus* from various dairy products and identified five *Lactobacillus fermentum* and two *Lactobacillus acidophilus* strains with high oxalate-degrading capacity. Further analysis revealed that these strains were resistant to acid, bile salts, and tolerant to multiple antibiotics, suggesting *lactobacilli* as a promising strategy for preventing urinary stone formation. Mehra and Viswanathan (2021) and Mehra et al. (2022) confirmed this potential by applying two probiotic strains, *Lactobacillus paragasseri* UBLG-36 and *Lactobacillus paracasei* UBLPC-87, to a rat kidney stone model. Rats pretreated with these probiotics showed reduced urinary oxalate, lower serum urea nitrogen and creatinine levels, decreased stone formation, and reduced renal histological damage. These findings suggest that *Lactobacillus paragasseri* UBLG-36 and *Lactobacillus paracasei* UBLPC-87 can be incorporated into functional foods to reduce oxalate excretion, alleviate renal oxidative stress, and prevent calcium oxalate crystal formation.

Further studies indicate that the reduction in kidney crystals by *Lactobacillus plantarum* N-1 may occur through modulation of arginine metabolism, and supplementation with *L. plantarum* N-1 increases the abundance of beneficial bacteria, improves intestinal inflammation, and enhances intestinal barrier function (Liu et al., 2021b; Wei et al., 2021). Rats receiving prophylactic treatment with *L. plantarum* J-15 showed reduced urinary oxalate, endotoxins, pro-inflammatory factors, and prostaglandins, alongside improvements in enteritis and intestinal barrier function, attributed to reduced serum lipopolysaccharide levels and modulation of TLR4/NF-κB/COX-2 signaling (Tian et al., 2022). These findings suggest that *Lactobacillus* can prevent hyperoxaluria by modulating the gut microbiota and enhancing intestinal barrier function. However, some studies have found no reduction in urinary oxalate excretion or plasma oxalate concentrations with *lactobacilli* supplementation, possibly due to strain-specific effects or the limited impact of single bacterial strains compared to the gut microbiome as a whole (Siener et al., 2013a). Clinical studies have reported that treatment with high concentrations of freeze-dried lactic acid bacteria (*L. acidophilus*, *L. plantarum*, *L. brevis*, *S. thermophilus*, and *B. infantis*) markedly reduced urinary oxalate excretion (Campieri et al., 2001). Notably, most research on *Lactobacillus* has been limited to *in vitro* and animal studies, indicating a potential gap between in vitro and *in vivo* oxalate degradation capabilities. Further investigations in controlled human trials are needed to assess the efficacy of *lactobacilli* as oxalate-degrading probiotics.

### 3.3 Bifidobacteria

*Bifidobacteria* are anaerobic, Gram-positive bacilli often isolated from infant feces (Yao et al., 2021). Chen J. et al. (2021) reported that *bifidobacteria* gently modulate the gut microbiota while exhibiting antibacterial, antioxidative stress, and immunoregulatory properties, making them popular ingredients in functional foods. In addition to promoting gut health, *Bifidobacteria* influence various organs in the body through metabolic products of the gut microbiota (Zhang et al., 2024).

Recent studies have shown that *Bifidobacteria* can reduce urinary oxalate levels and prevent kidney stone formation. Klimesova et al. (2015) used a high-oxalate mouse model to show that *Bifidobacteria* can colonize in vivo and degrade dietary oxalates, reducing urinary oxalate excretion and limiting intestinal absorption, thus aiding in the prevention of kidney stone formation. Masoleh et al. (2023) studied a probiotic blend containing *Bifidobacterium longum* and found that it inhibited oxalate production in a rat kidney stone model, reducing urinary oxalate levels. Jung et al. (2023) reported that six idiopathic calcium oxalate stone patients with hyperoxaluria experienced a significant reduction in 24-h urinary oxalate excretion after 4 weeks of daily intake of a *bifidobacteria*-containing mixture. These studies support the role of *bifidobacteria* in oxalate degradation and the prevention of calcium oxalate stones.

Clinical studies have found that probiotic mixtures containing *Lactobacillus* and *Bifidobacterium* can reduce urinary oxalate levels and the prevalence of crystalluria in patients with kidney stones to some extent (Vittori et al., 2024), but the effect is less pronounced, possibly due to the complex interactions between the host diet, gut microbiota, and oxalate-degrading activity across different bacterial species (Tavasoli et al., 2021). Therefore, further research is needed to determine the optimal combinations of *bifidobacteria* and other probiotics for effective prevention of kidney stones.

## 4 Other probiotics and recombinant probiotics associated with oxalate metabolism

### 4.1 Enterococcus faecalis

*Enterococcus faecalis* is a Gram-positive, facultative anaerobic bacterium commonly found in the human intestinal microbiota. While it serves as a beneficial microorganism in the gut, it can also act as an opportunistic pathogen, contributing to biofilm-associated infections, particularly in the gastrointestinal and urinary tracts (Govindarajan et al., 2022). Hokama et al. (2005) demonstrated that *E. faecalis* degrades oxalic acid through three specific proteins, with molecular weights of 65, 48, and 40 kDa. These proteins were absent when the strain lost its oxalate-degrading capacity, highlighting their crucial role in the degradation process and suggesting they could be key targets for future research. The absence of these proteins under conditions with alternative energy sources further suggests that oxalate degradation may act as a compensatory mechanism in unfavorable conditions (García-Solache and Rice, 2019). Recent studies have also revealed interactions between *E. faecalis* and the host immune system, modulating inflammatory responses and immune cell activities, which may reduce inflammation associated with kidney stone formation (Wang et al., 2008). Further investigation of this interaction could lead to novel strategies for preventing and treating renal calculi. However, large-scale, randomized controlled trials have not yet validated the safety or efficacy of *E. faecalis* as a probiotic, and as such, enterococcus are not yet approved for clinical use in treating or improving human diseases (Wang et al., 2020).

### 4.2 Faecalibacterium

*Faecalibacterium* is a Gram-positive bacterium in the human gut, with its core member *F. prausnitzii* being an important butyrate-producing bacterium in the intestinal microbiota, playing a key role in maintaining gut health, metabolic regulation, and immune function (Martín et al., 2023). 16S rRNA sequencing has revealed that the relative abundance of *Faecalibacterium* is reduced in the gut microbiota of patients with calcium oxalate stones and is negatively correlated with stone formation (Ticinesi et al., 2018). This indicates that lower abundance of *Faecalibacterium* and *F. prausnitzii* is associated with a higher risk of kidney stone formation (Choy et al., 2024). Related studies administering *Faecalibacterium prausnitzii* to mice on a high-oxalate diet demonstrated that it alleviated renal calcium oxalate crystal deposition by reducing the production of deoxycholic acid and secondary bile acids (Liu et al., 2025). Although *Faecalibacterium* is not a specialized “oxalate-degrading bacterium,” it can improve the intestinal environment through butyrate, thereby indirectly enhancing the stability of intestinal oxalate metabolism (Chen F. et al., 2021).

### 4.3 Lachnospira

*Lachnospira* exhibits strict anaerobic metabolic characteristics. This genus can ferment complex carbohydrates such as pectin and polygalacturonic acid to produce short-chain fatty acids (SCFAs), including acetate and formate, making it a typical SCFA-producing bacterial group (Cornick and Stanton, 2015). In the aforementioned study by Suryavanshi, the proportion of oxalate-degrading species was higher in stone formers (Suryavanshi et al., 2016). Therefore, a high intake of oxalate-rich foods can lead to specific alterations in the gut microbiota composition, including increased relative abundance of *Lachnospira*, *Roseburia*, *Dialister*, *Faecalibacterium*, and *Lactobacillus*, accompanied by enhanced SCFA production (Ticinesi et al., 2020). To explore the relationship between gut microbiota and SCFAs in calcium oxalate nephrolithiasis, sequencing studies have shown that normal controls had higher levels of bacteria such as *Lachnospiraceae*, *Ruminococcus*, and *Anaerostipes* compared with kidney stone patients; these bacteria commonly produce SCFAs as metabolic products (Oliphant and Allen-Vercoe, 2019). SCFAs can also enter the host circulation and reach the kidneys, where they improve inflammation and fibrosis progression in chronic kidney disease (Li et al., 2017). Moreover, *in vivo* studies have demonstrated that SCFAs such as acetate, propionate, and butyrate can reduce renal calcium oxalate stone formation in model rats (Liu Y. et al., 2020).

### 4.4 *Eubacterium* genus

The genus *Eubacterium* consists of anaerobic, Gram-positive bacilli that play a significant role in oxalate metabolism and are key components of the gut microbiota. These species contribute to maintaining intestinal function, modulating inflammation, and regulating immune responses (Mukherjee et al., 2020). Ito et al. (1996) isolated Lentil WHY-1 from a male fecal sample and demonstrated its ability to degrade oxalate in artificial intestinal fluid. Its capacity to proliferate and metabolize oxalate in the presence of bile salts highlights the clinical potential of *Eubacterium* species. Additionally, studies have shown that SCFAs produced by various *Eubacterium* species significantly contribute to human health by providing nutrients and energy for the gut epithelium, maintaining the mucosal barrier, and alleviating inflammation (Louis and Flint, 2017; Zhou et al., 2023). Further research indicates that SCFAs can enhance the expression of the intestinal oxalate transporter SLC26A6, promoting oxalate secretion and reducing absorption, suggesting that SCFAs may influence kidney stone formation through multiple mechanisms (Liu et al., 2021a). Consequently, several *Eubacterium* species are being considered for trials as next-generation probiotics. However, there is currently no direct evidence confirming their oxalate-degrading abilities in vivo, and additional studies are needed to explore their potential in preventing and managing calcium oxalate kidney stones.

## 5 Recombinant probiotics for oxalate metabolism

As discussed earlier, probiotics hold significant promise for the prevention and treatment of renal calcium oxalate stones. However, not all probiotics are capable of degrading oxalate. Each probiotic strain has specific environmental requirements and functional properties, with those harboring one or more oxalate-degrading enzymes—such as oxalate decarboxylase (OxdC), Frc, or Oxc—demonstrating superior oxalate degradation capabilities. Given the vast diversity of probiotics, extensive screening is necessary to identify strains containing these enzymes. In addition to oxalate degradation, strategies aimed at inhibiting inflammatory factor production and alleviating oxidative stress have shown effectiveness in mitigating hyperoxaluria (Liu X. et al., 2020). Therefore, combining oxalate-degrading enzymes with *Lactobacillus* strains possessing anti-inflammatory and antioxidative properties may offer a novel therapeutic approach. Sasikumar et al. (2014b) successfully transferred the recombinant vector pLdhl0373OxdC into *Lactobacillus* WCFS1, resulting in the recombinant strain WCFS1-OxdC. OxdC activity was detected in the culture supernatant of the recombinant strain, leading to a 70% reduction in oxalate levels, while no reduction was observed in the supernatant of wild-type *Lactobacillus* WCFS1. Furthermore, when administered to rats fed a 5% potassium oxalate diet to induce calcium oxalate stones, WCFS1-OxdC significantly reduced urinary oxalate levels, while wild-type WCFS1 had no effect (Sasikumar et al., 2014a). Zhao et al. (2018) introduced genes encoding both oxalate decarboxylase and oxalate oxidase into *Lactococcus lactis* MG1363, resulting in the recombinant strain MG1363-OxdC. This strain exhibited the ability to degrade oxalate *in vitro* and effectively reduced oxalate levels in both the medium and rat urine when administered orally to a rat model of hyperoxaluria. Similarly, Paul et al. (2018) developed a food-grade recombinant *Lactobacillus plantarum* that secreted OxdC, which lowered urinary oxalate levels and reduced calcium deposits in rat kidneys. Collectively, these studies highlight the broad potential of recombinant *lactobacilli* as a next-generation therapeutic approach, offering alternative strategies for preventing calcium oxalate kidney stones.

*Bacillus subtilis* is a versatile bacterium capable of taking up extracellular DNA, facilitating genetic modifications (Li et al., 2021). The oxalate decarboxylase derived from *B. subtilis* has shown potential as a therapeutic agent for calcium oxalate urolithiasis (Albert et al., 2017). The integration of the *B. subtilis*-derived oxalate decarboxylase gene into bacterial strains through targeted chromosomal mutagenesis, using the mobile genetic element Ll.LtrB, has demonstrated the ability to degrade extracellular oxalate (Paul et al., 2019). Pfau et al. (2021) conducted a clinical study using a recombinant oxalate decarboxylase, reloxaliase, derived from *B. subtilis*, and found that reloxaliase effectively reduced plasma oxalate concentrations in patients with enteric hyperoxaluria, thereby decreasing the risk of oxalate-related diseases. Furthermore, Lingeman et al. (2019) developed an oral enzyme formulation, ALLN-177, for the treatment of severe hyperoxaluria by expressing, purifying, and encapsulating oxalate decarboxylase from *Bacillus subtilis*. In a clinical trial involving 16 subjects with hyperoxaluria and a history of kidney stones, ALLN-177 significantly reduced 24-h urinary oxalate excretion and exhibited good tolerability. Collectively, these studies suggest that genetically engineered *B. subtilis* strains capable of degrading oxalate could serve as a promising adjunctive method for preventing and treating calcium oxalate nephrolithiasis.

## 6 Discussion

Probiotics are integral components of the human gut microbiome, closely linked to the development and progression of kidney stones. Significant advancements have been made in investigating the prophylactic and therapeutic roles of probiotics in the formation of renal calcium oxalate stones. Several oxalate-degrading probiotics that influence stone formation have been identified. However, most research has focused on *Oxalobacter formigenes*, a bacterium that is susceptible to antibiotics, which limits its clinical utility. To develop more effective probiotic interventions for preventing and treating calcium oxalate stones, gene recombination to introduce oxalate-degrading enzymes into well-colonizing *Lactobacillus* strains in the gut may represent a promising new approach. It has been reported that the intestinal microbiota collectively influences the development of renal calcium oxalate stones (Ticinesi et al., 2019). Therefore, probiotic interventions aimed at modulating intestinal oxalate absorption must account for the complexity of the gut microbial community.

Future studies must address the limitations of previous research. Historically, animal experiments have primarily involved probiotic supplementation and dietary modifications to modulate the gut microbiota in rodents. However, these approaches fail to fully replicate human intestinal conditions, necessitating the use of more representative animal models to validate efficacy. Additionally, experiments linking microbial populations, functional oxalate degradation, molecular analyses of related gene regulation, and bacterial survival in the gut are crucial for identifying probiotics suitable for treating renal stone disease. The safety and long-term efficacy of these probiotics for preventing and treating renal oxalate stones must be confirmed in both healthy subjects and patients with kidney stones. An increasing number of clinical trials are providing strong scientific evidence for the application of probiotics in medical practice. With continued exploration and research, probiotics hold the potential to become an ideal novel strategy for the prevention and treatment of calcium oxalate nephrolithiasis.

## 7 Conclusion

Probiotics, particularly recombinant strains, show considerable potential in the prevention and treatment of calcium oxalate kidney stones. By modulating gut microbiota and enhancing oxalate degradation, probiotics can reduce the absorption of oxalate, thereby preventing stone formation. Despite promising results, further clinical studies and *in vivo* research are needed to validate their efficacy and optimize therapeutic strategies. Probiotics offer an innovative, non-invasive approach for the management and recurrence prevention of kidney stones.
